# Efficacy and Safety of Dapagliflozin in Patients With CKD Across Major Geographic Regions

**DOI:** 10.1016/j.ekir.2022.01.1060

**Published:** 2022-02-02

**Authors:** Priya Vart, Ricardo Correa-Rotter, Fan Fan Hou, Niels Jongs, Glenn M. Chertow, Anna Maria Langkilde, John J.V. McMurray, Peter Rossing, C. David Sjöström, Bergur V. Stefansson, Robert D. Toto, Walter Douthat, Elizabeth Escudero, Rey Isidto, Dinesh Khullar, Harpreet S. Bajaj, David C. Wheeler, Hiddo J.L. Heerspink

**Affiliations:** 1 Department of Clinical Pharmacy and Pharmacology, University Medical Center Groningen, University of Groningen, Groningen, The Netherlands; 2 The National Medical Science and Nutrition Institute Salvador Zubiran, Mexico City, Mexico; 3 Division of Nephrology, Department of Medicine, Southern Medical University, National Clinical Research Center for Kidney Disease, Guangzhou, China; 4 Department of Medicine, Stanford University School of Medicine, Stanford, California, USA; 5 Department of Epidemiology and Population Health, Stanford University School of Medicine, Stanford, California, USA; 6 Late-Stage Development, Cardiovascular, Renal, and Metabolism, BioPharmaceuticals R&D, AstraZeneca, Gothenburg, Sweden; 7 Institute of Cardiovascular and Medical Sciences, University of Glasgow, Glasgow, UK; 8 Steno Diabetes Center Copenhagen, Gentofte, Denmark; 9 Department of Clinical Medicine, University of Copenhagen, Copenhagen, Denmark; 10 Department of Internal Medicine, UT Southwestern Medical Center, Dallas, Texas, USA; 11 Department of Nephrology, Hospital Privado Universitario de Cordoba, Cordoba, Argentina; 12 Division of Nephrology, Hospital Arzobispo Loayza, Cayetano Heredia University, Lima, Peru; 13 Healthlink Medical, Dental, Surgical Clinics and Diagnostics Center, Iloilo City, Philippines; 14 Department of Nephrology and Renal Transplant Medicine, Max Super Speciality Hospital, Saket, New Delhi, India; 15 LMC Diabetes and Endocrinology, Brampton, Ontario, Canada; 16 Department of Renal Medicine, University College London, London, UK; 17 The George Institute for Global Health, Sydney, Australia

**Keywords:** dapagliflozin, efficacy, regions, safety, SGLT-2 inhibitor

## Abstract

**Introduction:**

This study aimed to examine the efficacy and safety of dapagliflozin in the Dapagliflozin and Prevention of Adverse Outcomes in Chronic Kidney Disease (DAPA-CKD) trial (NCT03036150) by geographic region.

**Methods:**

Adults with chronic kidney disease (CKD) with or without type 2 diabetes, with estimated glomerular filtration rate (eGFR) 25 to 75 ml/min per 1.73 m^2^ and urinary albumin-to-creatinine ratio (UACR) 200 to 5000 mg/g were randomized to dapagliflozin (10 mg once daily) or placebo. The primary end point was a composite of a sustained decline in eGFR of ≥50%, end-stage kidney disease or death from kidney or cardiovascular causes. We categorized recruiting countries into 4 broad global regions: Asia, Europe, Latin America, and North America. Of 4304 randomized patients, 1346 (31.3%) were from Asia, 1233 (28.6%) from Europe, 912 (21.2%) from Latin America, and 813 (18.9%) from North America.

**Results:**

The relative risk of the primary composite end point was lower in patients randomized to dapagliflozin (relative to placebo) in all regions, with hazard ratios (95% CI) of 0.70 (0.48–1.00), 0.60 (0.43–0.85), 0.61 (0.43–0.86), and 0.51 (0.34–0.76) among patients from Asia, Europe, Latin America, and North America, respectively. There was no effect modification by region (interaction *P* = 0.77). Occurrence of serious adverse events (SAEs) was lower among patients randomized to dapagliflozin versus placebo (21.9% vs. 26.8%, 34.1% vs. 38.6%, 29.8% vs. 31.5%, and 34.9% vs. 41.0% in Asia, Europe, Latin America, and North America, respectively).

**Conclusion:**

Dapagliflozin reduced kidney and cardiovascular events and prolonged survival in patients with CKD, with and without type 2 diabetes, with no apparent effect modification by geographic region.

## Graphical abstract

**Figure:**
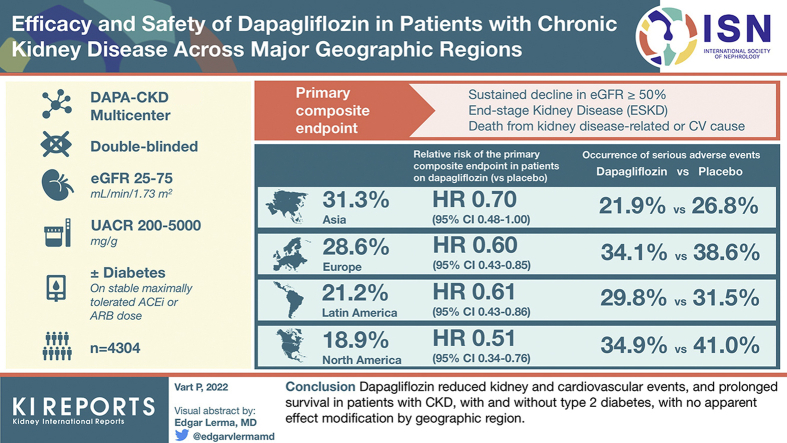

In the past 2 decades, there has been a considerable increase in the number of global clinical trials with a sizeable increase in the recruitment of patients from developing countries.^1^ This trend has also been noted in trials of cardiovascular and CKD.^2^ Regional differences in patient characteristics, comorbidities, and medical practice^3^^,^^4^ may result in differences in the efficacy and safety profiles of a drug across regions.

Recently, several large clinical trials investigating sodium-glucose cotransporter 2 inhibitors, initially developed for the treatment of hyperglycemia in type 2 diabetes, have shown favorable effects on kidney and cardiovascular outcomes in different patient populations.^5^, ^6^, ^7^ Some of these studies reported regional differences in efficacy. The Empagliflozin Outcome Trial in Patients with Chronic Heart Failure and a Reduced Ejection Fraction (EMPEROR-Reduced) trial reported the most pronounced effects on the composite outcome of heart failure hospitalization and cardiovascular death in patients from Asia and least pronounced effects in patients from Europe.^7^ These differences were also evident in a meta-analysis of 2 trials of sodium-glucose cotransporter 2 inhibitors in heart failure with reduced ejection fraction.^8^

The DAPA-CKD trial enrolled patients from 4 geographic regions including Asia, Europe, Latin America, and North America. In the present study, we investigated whether there were meaningful regional differences in efficacy and safety of dapagliflozin among patients with CKD with and without type 2 diabetes.

## Methods

### Study Design and Participants

DAPA-CKD was a randomized, double-blind, placebo-controlled multicenter trial conducted at 386 study sites in 21 countries (broadly categorized into 4 regions—Asia, Europe, Latin America, and North America) from February 2017 until June 2020. Manuscripts describing details of the study design and the primary results have been published previously.^9^, ^10^, ^11^, ^12^ Participants were adults aged 18 years or older with CKD with or without type 2 diabetes, with eGFR 25 to 75 ml/min per 1.73 m^2^ and UACR 200 to 5000 mg/g. Patients with type 1 diabetes, polycystic kidney disease, lupus nephritis, or antineutrophil cytoplasmic autoantibody-associated vasculitis as well as those receiving immunotherapy for primary or secondary kidney disease within 6 months before enrolment were excluded. All eligible patients were required to be treated with a stable maximally tolerated dose of an angiotensin-converting enzyme inhibitor or angiotensin receptor blocker for ≥4 weeks before randomization unless there was a documented intolerance to these drugs. All participants provided signed informed consent before the start of any study-related procedure. The trial was sponsored by AstraZeneca, and the trial protocol was approved by a central or local ethics committee at each trial site. The trial was registered at ClinicalTrials.gov (NCT03036150).

### Procedures

Eligible participants were randomly assigned to receive dapagliflozin 10 mg once daily or a matching placebo, in line with the sequestered, fixed-randomization schedule. Randomization was stratified by diabetes status and UACR (≤1000 or >1000 mg/g) at baseline. After randomization, in-person study visits were conducted after 2 weeks, 2, 4, and 8 months, and at 4-month intervals thereafter. At each follow-up visit, information on vital signs was recorded, blood and urine samples were obtained, and information on potential study end points, adverse events (AEs), concomitant therapies, and study drug adherence were collected.

### Regions

We broadly categorized participating sites into 4 geographic regions: Asia (participating countries: China, India, Japan, Philippines, South Korea, and Vietnam), Europe (participating countries: Denmark, Germany, Hungary, Poland, Russia, Spain, Sweden, United Kingdom, Ukraine), Latin America (participating countries: Argentina, Brazil, Mexico, Peru), and North America (participating countries: Canada, United States of America).

### Efficacy End Points

The primary end point was a composite of sustained ≥50% decline in eGFR (confirmed by a second serum creatinine after at least 28 days), the onset of end-stage kidney disease (defined as maintenance dialysis for >28 days, kidney transplantation, or eGFR <15 ml/min per 1.73 m^2^ confirmed by a second measurement after at least 28 days), or death from kidney or cardiovascular causes. Secondary end points were, in a hierarchical order, the following: a composite kidney end point of ≥50% sustained decline in estimated GFR, kidney failure, or death from kidney disease; a composite cardiovascular end point of cardiovascular death or hospitalization for heart failure; and all-cause mortality. All efficacy end points were adjudicated by an independent event adjudication committee using rigorous prespecified end point definitions.

### Safety

Given the extensive prior experience with dapagliflozin, ascertainment of AEs was limited to SAEs, AEs resulting in the discontinuation of study drug, and AEs of special interest (symptoms of volume depletion, kidney disease events, major hypoglycemia, bone fractures, amputations, potential diabetic ketoacidosis). Potential diabetic ketoacidosis events were adjudicated by an independent adjudication committee.

### Statistical Analysis

The overall analytical approach and prespecified statistical analysis plan have been previously published.^9^, ^10^, ^11^, ^12^ Briefly, all analyses presented here followed the intention-to-treat principle. For analysis of primary and secondary end points, we performed time-to-event analyses using a proportional hazards (Cox) regression stratified by randomization factors (diabetes status and UACR) and adjusting for baseline eGFR. We present corresponding hazard ratios and 95% CIs from model parameter coefficients and standard errors, respectively. To evaluate for effect modification by region, we included a multiplicative interaction term between randomized treatment and region. Where possible, treatment efficacy was investigated among patients with and without diabetes separately within each region. We assessed for nonuniformity of hazard ratios with Akaike’s information criterion.

We considered 2-tailed *P* < 0.05 to indicate statistical significance. We performed all analyses with Stata version 14.2 (Stata Corp).

## Results

Of 4304 randomized patients, 1346 (31.3%) were from Asia, 1233 (28.6%) from Europe, 912 (21.2%) from Latin America, and 813 (18.9%) from North America. Baseline characteristics of randomized patients stratified by regions are presented in Table 1. The mean age was slightly lower among patients in Asia and higher among patients in North America compared with patients in Europe and Latin America. The mean Quételet index (body mass index) was lowest in Asia and highest in North America. The level of systolic blood pressure and duration of diabetes was lower among patients in Asia compared with patients in other major geographic regions. The proportion of patients with diabetes was highest in North America. eGFR was similar across regions, but UACR was lower in North America and higher in Latin America. Prevalence of cardiovascular disease was lowest among patients in Asia. A similar proportion of patients were on angiotensin-converting enzyme inhibitor/angiotensin receptor blocker across regions.

**Table 1:** Characteristics of the patients at baseline according to major geographic region and randomized treatment assignment

Characteristic	Asia (*N* = 1346)		Europe (*N* = 1233)		Latin America (*N* = 912)		North America (*N* = 813)	
	Dapagliflozin	Placebo	Dapagliflozin	Placebo	Dapagliflozin	Placebo	Dapagliflozin	Placebo
	*n* = 692	*n* = 654	*n* = 610	*n* = 623	*n* = 449	*n* = 463	*n* = 401	*n* = 412
Age, years, mean (SD)	59.0 (12.5)	59.1 (12.8)	61.8 (12.3)	62.1 (11.7)	62.6 (10.8)	63.1 (11.5)	65.8 (11.0)	64.6 (11.6)
Female sex, *n* (%)	240 (34.7)	222 (33.9)	186 (30.5)	198 (31.8)	158 (35.2)	169 (36.5)	125 (31.2)	127 (30.8)
Race, *n* (%)								
Asian	692 (100)	654 (100)	12 (2.0)	7 (1.1)	4 (0.9)	7 (1.5)	41 (10.2)	50 (12.1)
Black or African American	0	0	2 (0.3)	4 (0.6)	33 (7.3)	24 (5.2)	69 (17.2)	59 (14.3)
Other	0	0	2 (0.3)	2 (0.3)	163 (36.3)	167 (36.1)	10 (2.5)	12 (2.9)
White	0	0	594 (97.4)	610 (97.9)	249 (55.5)	265 (57.2)	281 (70.1)	291 (70.6)
Body mass index (kg/m^2^), mean (SD)	25.7 (4.1)	25.5 (4.2)	30.8 (5.6)	31.5 (5.8)	29.8 (5.2)	29.9 (5.6)	33.2 (7.0)	33.2 (6.9)
Current Smoker, *n* (%)	111 (16.0)	102 (15.6)	93 (15.2)	100 (16.0)	35 (7.8)	56 (12.1)	44 (11.0)	43 (10.4)
Blood Pressure, mm Hg, mean, (SD)								
Systolic	132.5 (16.5)	133.2 (15.7)	138.9 (16.1)	140 (16.7)	140.5 (19.2)	141.8 (19.7)	136.6 (17.7)	134.9 (15.8)
Diastolic	77.1 (11.3)	77.8 (10.6)	79.1 (9.8)	78.8 (9.6)	78.4 (10.5)	78.3 (11.0)	74.8 (10.4)	74.2 (9.2)
eGFR, ml/min per 1.73 m^2^, mean (SD)	42.4 (11.1)	41.9 (11.3)	43.7 (12.4)	44.1 (12.5)	44.3 (13.8)	43.7 (13.4)	42.7 (12.2)	42.2 (12.5)
HbA1c, %, mean (SD)	6.9 (1.7)	6.8 (1.6)	6.8 (1.5)	6.9 (1.6)	7.6 (2.0)	7.4 (2.0)	7.3 (1.7)	7.2 (1.6)
Median UACR, mg/g (IQR)	984 (493–1854)	930 (492–1895)	1043 (467–1821)	914 (488–1608)	1089 (499–2273)	1061 (496–2099)	770 (412–1554)	869 (449–1915)
UACR >1000 mg/g, *n* (%)	342 (49.4)	312 (47.7)	314 (51.5)	291 (46.7)	240 (53.4)	243 (52.5)	152 (37.9)	185 (44.9)
Type 2 diabetes, *n* (%)	438 (63.3)	403 (61.6)	367 (60.2)	404 (64.8)	342 (76.2)	329 (71.1)	308 (76.8)	315 (76.5)
Median duration of diabetes, yr (IQR)	10.4 (5.7–17.3)	10.8 (5.6–19.1)	13.7 (7.3–19.3)	13.0 (7.4–20.4)	17.5 (9.7–24.7)	16.4 (9.4–22.9)	16.0 (8.2–22.5)	15.6 (8.9–22.1)
Cardiovascular disease, *n* (%)	165 (23.8)	157 (24.0)	307 (50.3)	288 (46.2)	167 (37.2)	173 (37.4)	174 (43.4)	179 (43.4)
Prior Medication, *n* (%)								
ACE inhibitor/ARB	678 (98.0)	637 (97.4)	606 (99.3)	614 (98.6)	444 (98.9)	459 (99.1)	384 (95.8)	387 (93.9)
Diuretic	152 (22.0)	149 (22.8)	329 (53.9)	341 (54.7)	223 (49.7)	235 (50.8)	224 (55.9)	229 (55.6)
Insulin^a^	205 (46.8)	183 (45.4)	194 (52.9)	213 (52.7)	227 (66.4)	186 (56.5)	188 (61.0)	202 (64.1)
DPP-4 inhibitors^a^	160 (36.5)	167 (41.4)	81 (22.1)	82 (20.3)	45 (13.2)	55 (16.7)	78 (25.3)	74 (23.5)
Biguanides^a^	144 (32.9)	120 (29.8)	178 (48.5)	202 (50.0)	187 (54.7)	167 (50.8)	122 (39.6)	124 (39.4)

ACE, angiotensin-converting enzyme; ARB, angiotensin receptor blocker; DPP-4, dipeptidyl peptidase 4; eGFR, estimated glomerular filtration rate; HbA1c, hemoglobin A1c; IQR, interquartile range; UACR, urinary albumin-to-creatinine ratio; USA, United States of America.

Participant countries from Asia: China *(N* = 210), India *(N* = 201), Japan *(N =* 244), Philippines *(N* = 115), South Korea (*N* = 294), Vietnam (*N* = 282). Participant countries from Europe: Denmark (*N* = 45), Germany (*N* = 138), Hungary (*N* = 140), Poland (*N* = 103), Russia (*N* = 255), Spain (*N* = 260), Sweden (*N* = 40), United Kingdom (*N* = 60), Ukraine (*N* = 192). Participant countries from Latin America: Argentina (*N* = 235), Brazil (*N* = 302), Mexico (*N* = 154), Peru (*N* = 221). Participants countries from North America: Canada (*N* = 280), USA (*N* = 533).

a In patients with diabetes.

### Primary End Point

**Figure 1:**
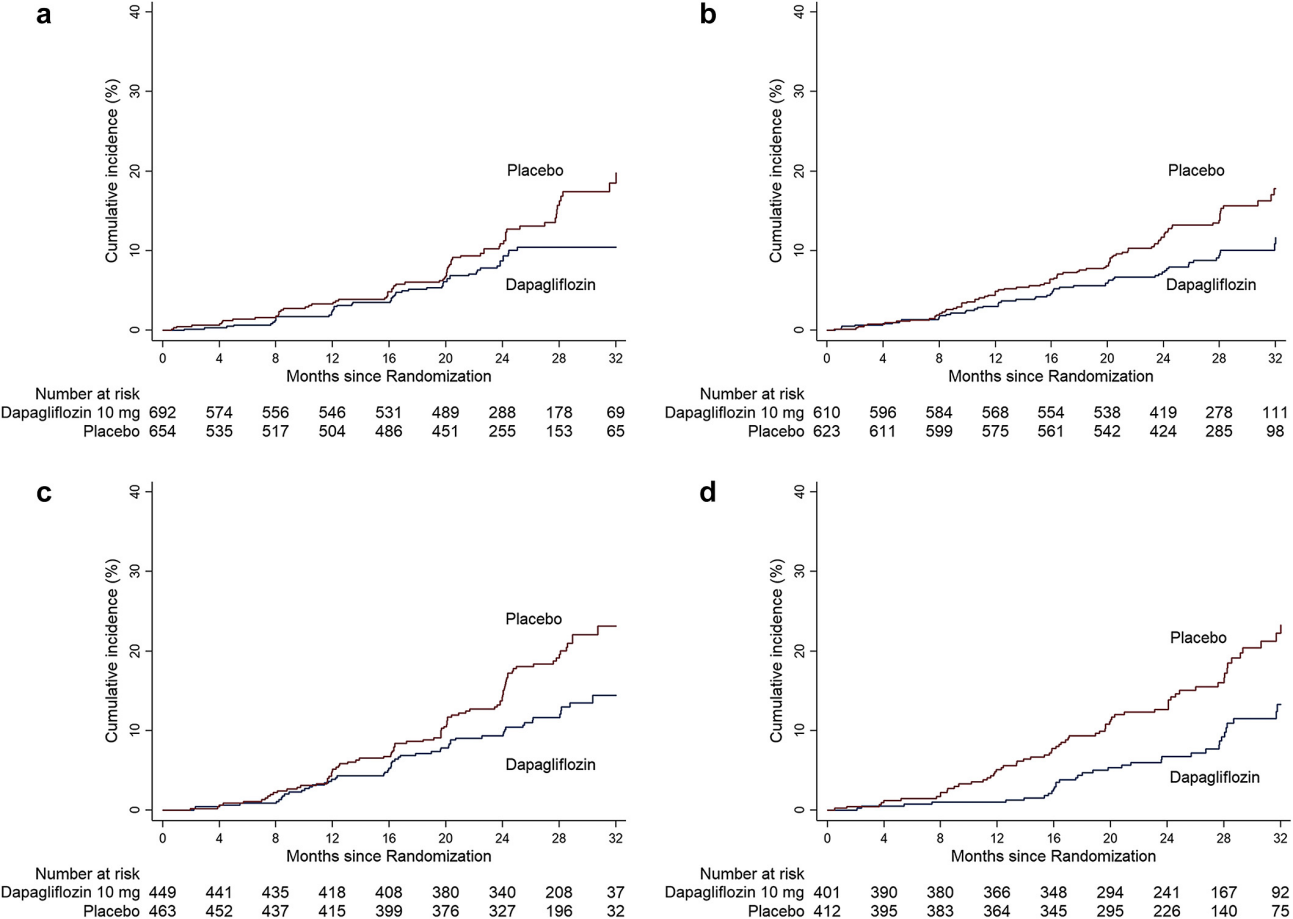
Cumulative incidence of primary end point by major geographic region. (a) Asia; (b) Europe; (c) Latin America; and (d) North America.

**Figure 2:**
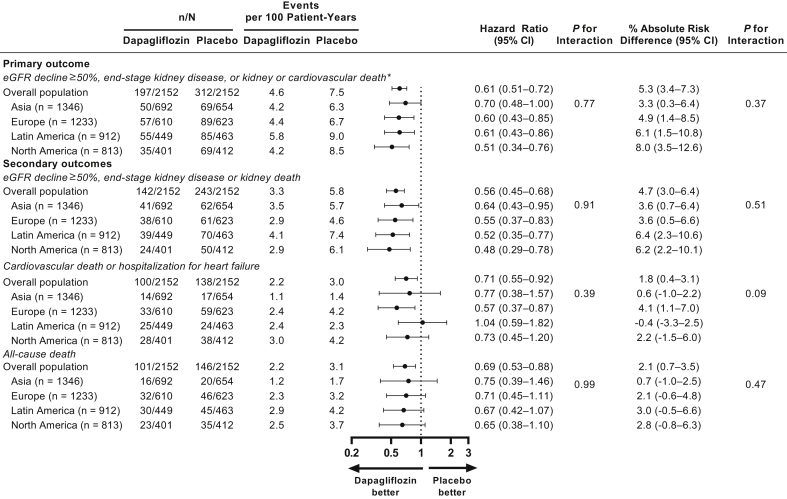
Efficacy of dapagliflozin for primary and key secondary end points by major geographic region. eGFR, estimated glomerular filtration rate.

Median follow-up duration was 1.9 years in Asia, 2.3 years in Europe and Latin America, and 2.1 years in North America. Event rates (per 100 patient-years) for the primary composite end point were 4.2, 4.4, 5.8, and 4.2 in patients randomized to dapagliflozin and 6.3, 6.7, 9.0, and 8.5 in patients randomized to placebo in Asia, Europe, Latin America, and North America, respectively. Figure 1a to d shows the cumulative incidence of the primary composite end point in both randomized groups, stratified by region. Relative risk reductions in the corresponding regions were the following: hazard ratio (95% CI) 0.70 (0.48–1.00), 0.60 (0.43–0.85), 0.61 (0.43–0.86), and 0.51 (0.34–0.76), respectively. Absolute risk reductions were 3.3% (0.3–6.4), 4.9% (1.4–8.5), 6.1% (1.5–10.8), and 8.0% (3.5–12.6) in Asia, Europe, Latin America, and North America, respectively. There was no evidence of heterogeneity of benefit on relative or absolute risk reductions by region (interaction *P* = 0.8 and 0.4, respectively) (Figure 2). Results were also consistent among patients with and without diabetes within each region (Supplementary Table S1). There was no apparent violation of the proportional hazards assumption.

### Secondary End Points

Similar to the primary end point, treatment with dapagliflozin led to a reduction in the incidence of the composite kidney end point, the composite cardiovascular end point, and all-cause death in patients across all regions. For all 3 secondary end points, there was no heterogeneity of benefit on relative or absolute risk reduction by region (Figure 2). Supplementary Figures S1A to D, S2A to D, and S3A to D show the cumulative incidence of the 3 secondary end points in both randomized groups, across the 4 designated regions. The number of events for secondary end points was insufficient to allow meaningful analyses by diabetes status within each region.

### AEs of Interest, SAEs, and Treatment Discontinuation

The number and proportions of SAEs, AEs of interest, and treatment discontinuation due to AEs observed in patients stratified by treatment group and region are presented in Table 2. SAEs were numerically less frequent in patients randomized to dapagliflozin (compared with placebo) within each region, and the difference in treatment and placebo group in SAEs was similar across regions.

**Table 2:** Safety by major geographic region

Outcome, *n* (%)	Dapagliflozin (*N* = 2149)	Placebo (*N* = 2149)	Odds ratio (95% CI)	*P* value interaction
Asia (*n* = 1344)	690	654		
Europe (*n* = 1229)	609	620		
Latin America (*n* = 912)	449	463		
North America (*n* = 813)	401	412		
Discontinuation due to adverse event				0.8
Overall	118 (5.5)	123 (5.7)	0.97 (0.74–1.26)	
Asia (*n* = 1344)	33 (4.8)	35 (5.3)	0.91 (0.56–1.49)	
Europe (*n* = 1229)	35 (5.7)	36 (5.8)	0.98 (0.60–1.58)	
Latin America (*n* = 912)	23 (5.1)	29 (6.3)	0.82 (0.46–1.44)	
North America (*n* = 813)	27 (6.7)	23 (5.6)	1.24 (0.69–2.22)	
Any serious adverse event^a^				0.8
Overall	633 (29.5)	729 (33.9)	0.81 (0.72–0.93)	
Asia (*n* = 1344)	151 (21.9)	175 (26.8)	0.77 (0.60–0.99)	
Europe (*n* = 1229)	208 (34.1)	239 (38.6)	0.82 (0.65–1.04)	
Latin America (*n* = 912)	134 (29.8)	146 (31.5)	0.92 (0.70–1.23)	
North America (*n* = 813)	140 (34.9)	169 (41.0)	0.78 (0.58–1.03)	
Adverse events of interest				
Amputation^b^				0.7
Overall	35 (1.6)	39 (1.8)	0.89 (0.56–1.41)	
Asia (*n* = 1344)	3 (0.4)	5 (0.8)	0.56 (0.13–2.35)	
Europe (*n* = 1229)	9 (1.5)	11 (1.8)	0.83 (0.34–2.02)	
Latin America (*n* = 912)	12 (2.7)	15 (3.2)	0.81 (0.37–1.76)	
North America (*n* = 813)	11 (2.7)	8 (1.9)	1.41 (0.56–3.56)	
Any definite or probable diabetic ketoacidosis				—
Overall	0	2 (0.1)		
Asia (*n* = 1344)	0	1 (0.1)	—	
Europe (*n* = 1229)	0	0	—	
Latin America (*n* = 912)	0	0	—	
North America (*n* = 813)	0	1 (0.2)	—	
Fracture^c^				0.7
Overall	85 (4.0)	69 (3.2)	1.25 (0.90–1.72)	
Asia (*n* = 1344)	22 (3.2)	21 (3.2)	1.00 (0.54–1.85)	
Europe (*n* = 1229)	21 (3.4)	19 (3.1)	1.12 (0.60–2.12)	
Latin America (*n* = 912)	20 (4.4)	13 (2.8)	1.62 (0.80–3.30)	
North America (*n* = 813)	22 (5.5)	16 (3.9)	1.45 (0.75–2.82)	
Renal related adverse event^c^				0.6
Overall	155 (7.2)	188 (8.7)	0.82 (0.65–1.02)	
Asia (*n* = 1344)	28 (4.1)	29 (4.4)	0.92 (0.54–1.57)	
Europe (*n* = 1229)	37 (6.1)	47 (7.6)	0.78 (0.50–1.22)	
Latin America (*n* = 912)	33 (7.3)	51 (11.0)	0.65 (0.41–1.03)	
North America (*n* = 813)	57 (14.2)	61 (14.8)	0.97 (0.65–1.44)	
Major hypoglycemia^d^				0.7
Overall	14 (0.6)	28 (1.3)	0.50 (0.26–0.95)	
Asia (*n* = 1344)	3 (0.4)	8 (1.2)	0.35 (0.09–1.33)	
Europe (*n* = 1229)	1 (0.2)	5 (0.8)	0.20 (0.02–1.75)	
Latin America (*n* = 912)	7 (1.6)	11 (2.4)	0.65 (0.25–1.71)	
North America (*n* = 813)	3 (0.7)	4 (1.0)	0.80 (0.18–3.64)	
Volume depletion^c^				0.4
Overall	127 (5.9)	90 (4.2)	1.44 (1.09–1.90)	
Asia (*n* = 1344)	27 (3.9)	18 (2.7)	1.44 (0.78–2.63)	
Europe (*n* = 1229)	35 (5.7)	34 (5.5)	1.05 (0.64–1.71)	
Latin America (*n* = 912)	21 (4.7)	13 (2.8)	1.70 (0.84–3.44)	
North America (*n* = 813)	44 (11.0)	25 (6.1)	1.92 (1.15–3.20)	

a Includes death.

b Surgical or spontaneous/nonsurgical amputation, excluding amputation due to trauma.

c Based on predefined list of preferred terms.

d Adverse event with the following criteria confirmed by the investigator: (i) symptoms of severe impairment in consciousness or behavior, (ii) need of external assistance, (iii) intervention to treat hypoglycemia, (iv) prompt recovery of acute symptoms after the intervention.

## Discussion

For any major international clinical trial, it is unlikely that patients from all participating regions or countries will have the same demographic characteristics, comorbidities, and background therapy. If regional differences are sizable, they have the potential to meaningfully influence the interpretation of clinical trial results vis-à-vis efficacy and safety within regions. In the current analysis, we aimed to determine whether there were regional differences in the effects of dapagliflozin on kidney and cardiovascular end points. We showed consistently favorable effects, despite differences in baseline characteristics and concomitant medication use, with substantial relative and absolute risk reductions of the primary composite end point and 3 secondary end points, including all-cause mortality. The safety profile of dapagliflozin was also similar across regions.

In line with our findings, other clinical trials using sodium-glucose cotransporter 2 inhibitors showed health benefits across regions. For instance, in the CREDENCE trial comparing canagliflozin and placebo in patients with type 2 diabetes and CKD (eGFR 30–90 ml/min per 1.73 m^2^), there was no regional heterogeneity in efficacy for the composite end point of a sustained decline in the estimated GFR of at least 50%, end-stage kidney disease, or death from renal or cardiovascular causes.^13^ Similarly, canagliflozin and ertugliflozin in patients with type 2 diabetes and increased risk of cardiovascular disease showed no regional heterogeneity in efficacy for a composite end point of cardiovascular death, myocardial infarction, or ischemic stroke.^14^^,^^15^

Regional differences in the efficacy of sodium-glucose cotransporter 2 inhibitors have been reported for patients with heart failure. In the EMPEROR-Reduced trial, the relative risk reduction for a composite end point of heart failure hospitalization and cardiovascular death was only 6% in Europe and was 45% in Asia.^7^ In EMPEROR-Reduced, the majority of events captured within the composite cardiovascular end point were hospitalized heart failure events. Effect estimates were no doubt influenced by the fact that acute events of heart failure exacerbation were more often treated in outpatient settings in Europe compared with other regions. In a time-to-event analysis of any composite end point, nonfatal events are counted before deaths. Therefore, the inclusion of a region where nonfatal events are less likely to be “counted” could result in an attenuated estimate of the treatment effect. Indeed, when nonfatal heart failure events that were treated in an outpatient setting were included in the analysis, the point estimate of benefit on the composite cardiovascular end point in Europe changed from 6% to 26% but was unchanged in other regions. In contrast, there was no regional variation in the effect of empagliflozin on cardiovascular mortality, the other component of the composite end point. Moreover, a previous study in patients with type 2 diabetes^16^ and in DAPA-CKD (wherein the contribution of heart failure hospitalization to the composite cardiovascular end point was similar in Europe and Asia), there was little to no regional variation in the effect of dapagliflozin on the composite cardiovascular end point.

Despite standardized inclusion and exclusion criteria, there were notable regional differences in the baseline clinical characteristics that could reflect biological differences, differences in access to healthcare, or other social determinants of health. Patients from Latin America had higher levels of albuminuria and systolic blood pressure, and patients from Asia had more favorable cardiovascular risk factor profiles (e.g., lower body mass index and systolic blood pressure and lower prevalence of diabetes and cardiovascular disease at baseline) compared with patients from other regions. These regional differences in baseline clinical characteristics may explain the increased incidence of the primary composite end point, secondary kidney composite end point, and all-cause mortality in Latin America and the reduced incidence of the composite cardiovascular end point and all-cause mortality in patients from Asia compared with other regions. Of note, compared with Europeans (a mostly White population), body mass index was lower in patients from Asia, but the prevalence of diabetes was similar. This phenomenon has been well described; the Asian population have a roughly similar risk of type 2 diabetes compared with White people, despite a lower body mass index.^17^

There were substantial regional differences in antidiabetic medication use. Insulin was used more frequently in Latin America and North America. Fewer patients with diabetes in Asia were treated with insulin and/or biguanides, and roughly twice as many patients in Asia were treated with dipeptidyl peptidase-4 inhibitors compared with patients in Europe or Latin America. This may indicate limited access to insulin and biguanides in Asia^18^ and/or may indicate a difference in prescription patterns between regions. Indeed, previous reports have suggested dipeptidyl peptidase-4 inhibitors to be more effective in improving glycemic levels in the Asian population than other populations,^19^^,^^20^ which may have prompted its use more often in Asia compared with other regions.

The strengths of the present analyses include the relatively large sample size from 4 major geographic regions, with the collection of detailed information on baseline clinical characteristics. Analysis was prespecified, and data were collected under a single protocol from all target regions. The protocol did not restrict or mandate the use of any cardiovascular or glucose-lowering medications with the exception that angiotensin-converting enzyme-inhibitors and angiotensin receptor blockers be used except when contraindicated. As such, the results are more generalizable across and within the geographic regions studied. There are also several limitations. The definition of regions was based on broad continental geography; only 21 countries were included, with only a few countries in each geographic region, and were largely high and upper-middle-income countries, limiting generalizability. In addition, interpreting differences across regions is difficult because they may reflect several influences other than geography, including race/ethnicity, genetics, cultural differences, diet and lifestyle, type of health care system, economics, and even climate and other environmental factors.

In conclusion, despite notable differences in baseline characteristics across regions, dapagliflozin reduced the risk of kidney and cardiovascular disease events and all-cause mortality in all regions, with no evidence of heterogeneity in efficacy or safety. These findings support the use of dapagliflozin in patients with CKD with and without type 2 diabetes across major regions around the world.

## Disclosure

RCR is a member of the Executive Committee of the DAPA-CKD study and has received grants/contracts from GlaxoSmithKline and Novo Nordisk, consulting fees from Boehringer Ingelheim and Chinook, and payment/honoraria as a speaker or advisor from Amgen, AstraZeneca, Bayer, Boehringer Ingelheim, Janssen, and Novo Nordisk. FFH is a member of the DAPA-CKD study executive committee and is a study investigator. She has received personal fees from AbbVie. GMC has received fees from AstraZeneca for the DAPA-CKD trial steering committee, research grants from NIDDK, and support for research staff attending meetings from Amgen; participated in data safety monitoring boards for Bayer and Recor; is on the board of directors for Satellite Healthcare and on trial steering committees for Akebia, Gilead, Sanifit, and Vertez; and holds stock options or stock options with Ardelyx, CloudCath, Durect, DxNow, Miromatrix, Outset, and Unicycive. AML, CDS, and BVS are employees and stockholders of AstraZeneca. JJVM has received payments to his employer, Glasgow University, for his work on clinical trials, consulting, and other activities from AstraZeneca, Cytokinetics, KBP Biosciences, Amgen, Bayer, Theracos, Ionis Pharmaceuticals, Dalcor Pharmaceuticals, Novartis, GlaxoSmithKline, Bristol Myers Squibb, Boehringer Ingelheim, Cardurion, and Alnylam, and has received personal lecture fees from Abbott, Alkem Metabolics, Eris Life Sciences, Hickma, Lupin, Sun Pharmaceuticals, Medscape/Heart.org, ProAdWise Communications, Radcliffe Cardiology, Servier, and the Corpus. PR has received honoraria to Steno Diabetes Center Copenhagen for steering group membership and/or lectures and advice from AstraZeneca, Novo Nordisk, Bayer, and Eli Lilly; advisory board participation from Sanofi Aventis and Boehringer Ingelheim; and steering group participation from Gilead. RDT received funding from AstraZeneca for participating in the steering committee for DAPA-CKD. He has received fees for consultancy from Boehringer Ingelheim, Reata Pharma, and Chinook Pharma. He has received honoraria for lectures from Medscape and Medical Education Resources. He has participated in DSMB or advisory boards for Bayer, Viofor, Akebia, and Otsuka. HSB has received honoraria for lectures from Eli Lilly, Novo Nordisk, and Medscape and received support for congress attendance from Novo Nordisk and AstraZeneca. DCW provides ongoing consultancy services to AstraZeneca and received personal fees from Bayer, Boehringer Ingelheim, Astellas, GlaxoSmithKline, Janssen, Napp, Mundipharma, Reata, Vifor Fresenius, and Tricida. HJLH has received funding/honoraria and consulting fees to his institution for Steering Committee membership and/or advisory board participation from AstraZeneca (DAPA-CKD study), AbbVie, Travere Pharmaceuticals, Janssen, Gilead, Bayer, Chinook, Merck, and CSL Pharma; consulting fees from Boehringer Ingelheim and Novo Nordisk; and honoraria for lectures from AstraZeneca; and has participated in advisory boards for Mitsubishi Tanabe and Mundipharma. All the other authors declared no competing interests.

## Supplementary Material

Supplementary File (PDF)

**Table S1.** Efficacy of dapagliflozin for the primary composite endpoint by major geographic region and type 2 diabetes status.

**Figure S1.** Cumulative incidence of a composite end point of ≥50% sustained decline in estimated GFR, kidney failure, or death from kidney disease by major geographic region.

**Figure S2.** Cumulative incidence of a composite end point of cardiovascular death or hospitalization for heart failure by major geographic region.

**Figure S3.** Cumulative incidence of all-Cause Mortality by major geographic region.

**CONSORT 2010 checklist of information to include when reporting a randomized trial.**
