# Supplementary material for: Efficacy and Safety of Dapagliflozin in Patients With CKD Across Major Geographic Regions
Source: Kidney Int Rep. 2022 Feb 2;7(4):699–707. doi: 10.1016/j.ekir.2022.01.1060 (PMC9039473; doi:10.1016/j.ekir.2022.01.1060)
Supplement: Supplementary File (PDF) [file mmc1.pdf]

Supplementary Information

Efficacy and Safety of Dapagliflozin in Patients with CKD Across Major Geographic Regions

Contents

Table S1: Table S1: Efficacy of dapagliflozin for primary endpoint by major geographic region and diabetes status

Figure S1: Cumulative incidence of a composite end point of ≥50% sustained decline in estimated GFR, kidney failure or death from kidney disease by major geographic region 2

Figure S2: Cumulative incidence of a composite end point of cardiovascular death or hospitalization for heart failure by major geographic region.. 4

Figure S3: Cumulative incidence of all-Cause Mortality by major geographic region.................. 5

CONSORT 2010 checklist of information to include when reporting a randomised trial.............5

Table S1: Efficacy of dapagliflozin for primary endpoint by major geographic region and diabetes status

	**Dapagliflozin (N=2,152)**		**Placebo (N=2,152)**		**Absolute Risk Difference (95% CI)**	**Hazard Ratio (95% CI)**
	No. (%)	Participants with Event/100 Patient-Yr	No. (%)	Participants with Event/100 Patient-Yr		
**Primary composite outcome**						
eGFR decline ≥50%, end-stage kidney disease, or kidney or cardiovascular death						
**Asia (n=1,346)**	**50 (7.2)**	**4.2**	**69 (10.6)**	**6.3**	**3.3% (0.3, 6.4)**	**0.70 (0.48, 1.00)**
Diabetes (n=841)	35 (8.0)	4.5	47 (11.7)	6.7	3.7% (-0.4, 7.7)	0.69 (0.44, 1.07)
Non-diabetes (n=505)	15 (5.9)	3.7	22 (8.8)	5.5	2.9% (-1.7, 7.4)	0.71 (0.36, 1.38)
**Europe (n=1,233)**	**57 (9.3)**	**4.4**	**89 (14.3)**	**6.7**	**4.9% (1.4, 8.5)**	**0.60 (0.43, 0.85)**
Diabetes (771)	40 (10.9)	5.1	56 (13.9)	6.5	3.0% (-1.7, 7.6)	0.74 (0.50, 1.12)
Non-diabetes (462)	17 (7.0)	3.2	33 (15.1)	7.1	8.1% (2.4, 13.8)	0.41 (0.23, 0.75)
**Ltn/Sth America(n=912)**	**55 (12.2)**	**5.8**	**85 (18.4)**	**9.0**	**6.1% (1.5, 10.8)**	**0.61 (0.43, 0.86)**
Diabetes (n=671)	48 (14.0)	6.5	71 (21.6)	10.4	7.5% (1.8, 13.3)	0.62 (0.43, 0.89)
Non-diabetes (241)	7 (6.5)	3.2	14 (10.4)	5.2	3.9% (-3.1, 10.9)	0.59 (0.24, 1.48)
**North America (n=813)**	**35 (8.7)**	**4.2**	**69 (16.7)**	**8.5**	**8.0% (3.5, 12.6)**	**0.51 (0.34, 0.76)**
Diabetes (623)	29 (9.4)	4.5	55 (17.5)	8.8	8.0% (2.7, 13.4)	0.53 (0.34, 0.84)
Non-diabetes (190)	6 (6.4)	3.2	14 (14.4)	7.4	8.0% (-0.6, 16.6)	0.37 (0.14, 1.00)

Figure S1: Cumulative incidence of a composite end point of ≥50% sustained decline in estimated GFR, kidney failure or death from kidney disease by major geographic region

A) Asia

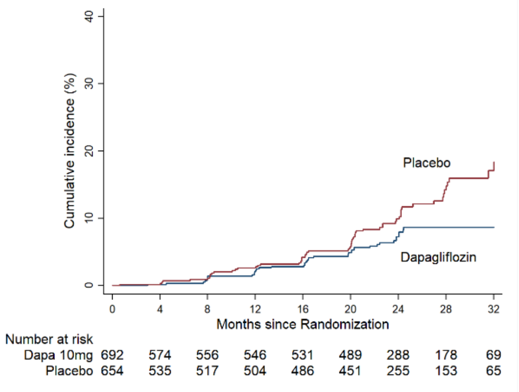

B) Europe

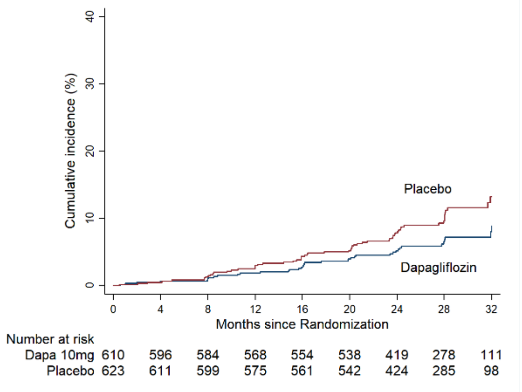

C) Latin America

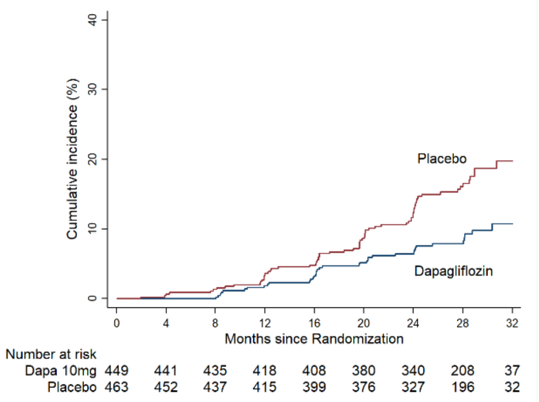

D) North America

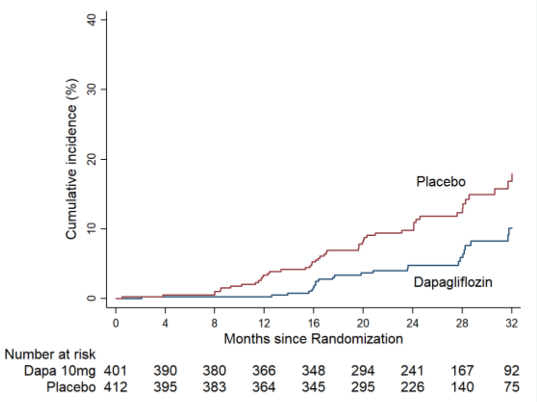

Figure S2: Cumulative incidence of a composite end point of cardiovascular death or hospitalization for heart failure by major geographic region

A) Asia

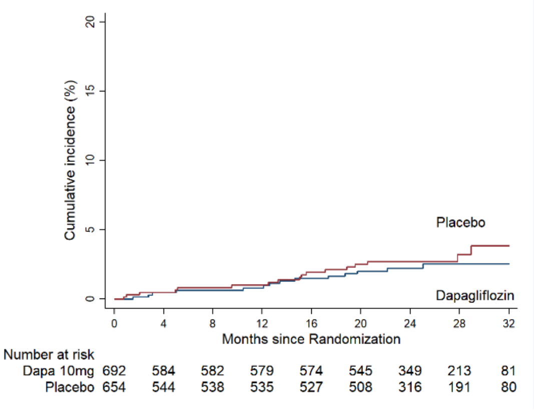

B) Europe

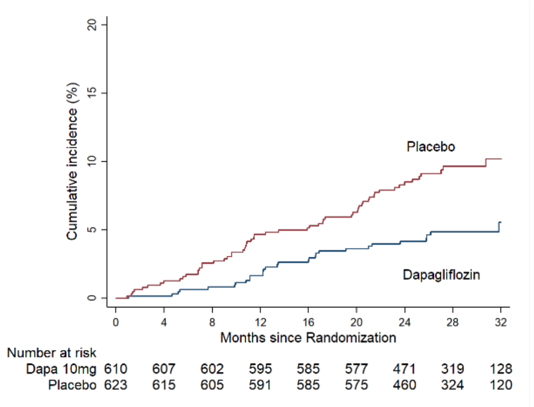

C) Latin America

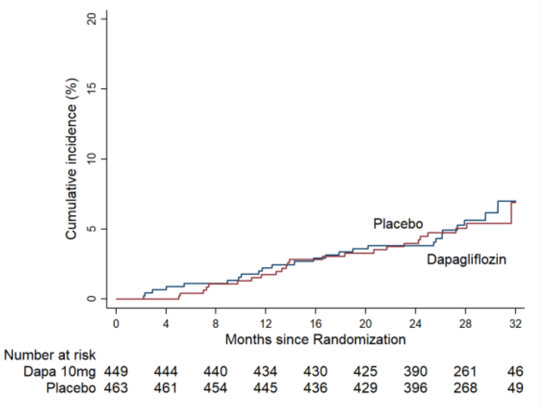

D) North America

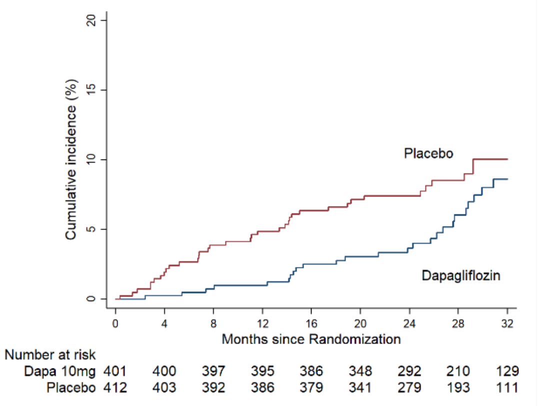

Figure S3: Cumulative incidence of all-Cause Mortality by major geographic region

A) Asia

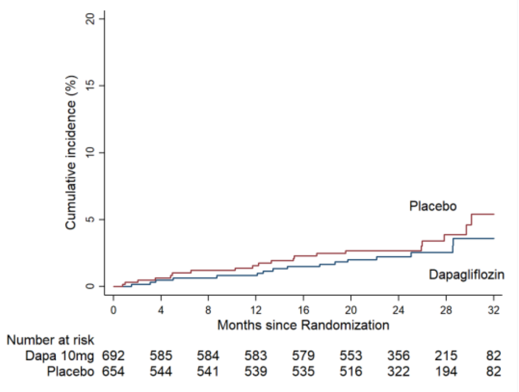

B) Europe

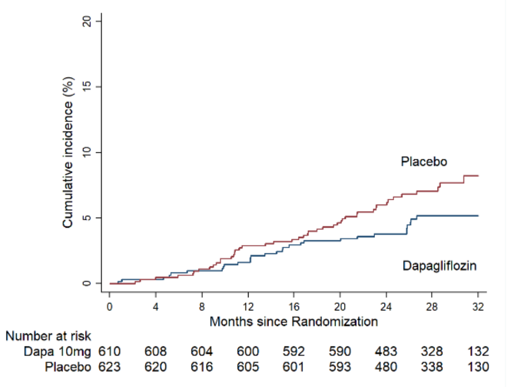

C) Latin America

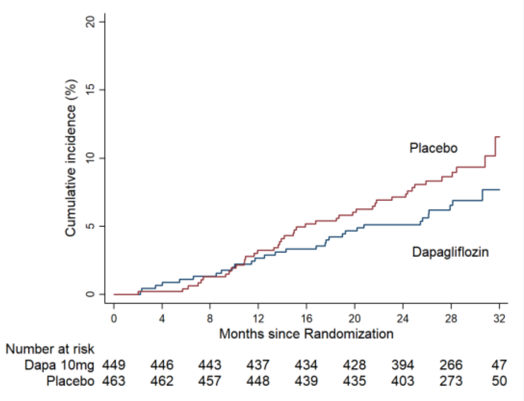

D) North America

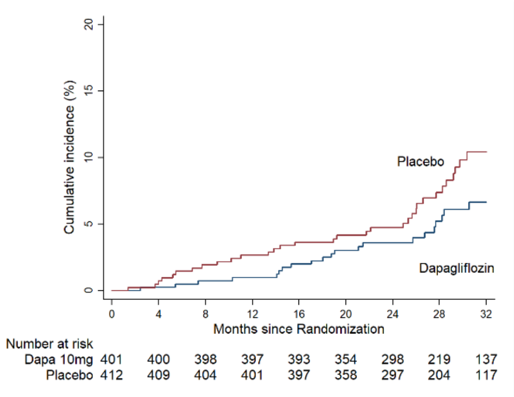

CONSORT 2010 checklist of information to include when reporting a randomised trial*

Section/Topic	Item No	Checklist item	Reported on page No
**Title and abstract**			
	1a	Identification as a randomised trial in the title	Page 1
	1b	Structured summary of trial design, methods, results, and conclusions (for specific guidance see CONSORT for abstracts)	Page 3
**Introduction**			
Background and objectives	2a	Scientific background and explanation of rationale	Page 5
	2b	Specific objectives or hypotheses	Page 5
**Methods**			
Trial design	3a	Description of trial design (such as parallel, factorial) including allocation ratio	Page 6 (refs 9-12)
	3b	Important changes to methods after trial commencement (such as eligibility criteria), with reasons	N/A
Participants	4a	Eligibility criteria for participants	Page 6
	4b	Settings and locations where the data were collected	Page 6,7
Interventions	5	The interventions for each group with sufficient details to allow replication, including how and when they were actually administered	Page 6-7 (refs 9-12)
Outcomes	6a	Completely defined pre-specified primary and secondary outcome measures, including how and when they were assessed	Page 7 (refs 9-12)
	6b	Any changes to trial outcomes after the trial commenced, with reasons	N/A
Sample size	7a	How sample size was determined	Page 6 (refs 9-12)
	7b	When applicable, explanation of any interim analyses and stopping guidelines	Previous paper (ref 9)
Randomisation:			

Sequence generation	8a	Method used to generate the random allocation sequence	Previous paper (ref 9)
	8b	Type of randomisation; details of any restriction (such as blocking and block size)	Previous paper (ref 9)
Allocation concealment mechanism	9	Mechanism used to implement the random allocation sequence (such as sequentially numbered containers), describing any steps taken to conceal the sequence until interventions were assigned	Previous paper (ref 9)
Implementation	10	Who generated the random allocation sequence, who enrolled participants, and who assigned participants to interventions	Previous paper (ref 9)
Blinding	11a	If done, who was blinded after assignment to interventions (for example, participants, care providers, those assessing outcomes) and how	Previous paper (ref 9)
	11b	If relevant, description of the similarity of interventions	N/A
Statistical methods	12a	Statistical methods used to compare groups for primary and secondary outcomes	Page 8
	12b	Methods for additional analyses, such as subgroup analyses and adjusted analyses	Page 8
**Results**			
Participant flow (a diagram is strongly recommended)	13a	For each group, the numbers of participants who were randomly assigned, received intended treatment, and were analysed for the primary outcome	Page 9
	13b	For each group, losses and exclusions after randomisation, together with reasons	Page 10, Table 2
Recruitment	14a	Dates defining the periods of recruitment and follow-up	Previous paper (Ref
	14b	Why the trial ended or was stopped	Previous paper (Ref
Baseline data	15	A table showing baseline demographic and clinical characteristics for each group	Page 22
Numbers analysed	16	For each group, number of participants (denominator) included in each analysis and whether the analysis was by original assigned groups	Table 1, 2, Figures 1,2

Outcomes and estimation	17a	For each primary and secondary outcome, results for each group, and the estimated effect size and its precision (such as 95% confidence interval)	Figure 2, page 9-10
	17b	For binary outcomes, presentation of both absolute and relative effect sizes is recommended	N/A
Ancillary analyses	18	Results of any other analyses performed, including subgroup analyses and adjusted analyses, distinguishing pre-specified from exploratory	Table 2, Figure 1,2, page 9-10
Harms	19	All important harms or unintended effects in each group (for specific guidance see CONSORT for harms)	Page 10
**Discussion**			
Limitations	20	Trial limitations, addressing sources of potential bias, imprecision, and, if relevant, multiplicity of analyses	Page 13-14
Generalisability	21	Generalisability (external validity, applicability) of the trial findings	Page 13-14
Interpretation	22	Interpretation consistent with results, balancing benefits and harms, and considering other relevant evidence	Page 11-14
**Other information**			
Registration	23	Registration number and name of trial registry	Page 3, 6
Protocol	24	Where the full trial protocol can be accessed, if available	Reference 9
Funding	25	Sources of funding and other support (such as supply of drugs), role of funders	Page 17

*We strongly recommend reading this statement in conjunction with the CONSORT 2010 Explanation and Elaboration for important clarifications on all the items. If relevant, we also recommend reading CONSORT extensions for cluster randomised trials, non-inferiority and equivalence trials, non-pharmacological treatments, herbal interventions, and pragmatic trials. Additional extensions are forthcoming: for those and for up to date references relevant to this checklist, see www.consort-statement.org.

REFERENCES

9. Heerspink HJL, Stefánsson BV, Correa-Rotter R, Chertow GM, Greene T, Hou FF, Mann JFE, McMurray JJV, Lindberg M, Rossing P, Sjöström CD, Toto RD, Langkilde AM, Wheeler DC; DAPA-CKD Trial Committees and Investigators. Dapagliflozin in Patients with Chronic Kidney Disease. N Engl J Med. 2020 Oct 8;383(15):1436-1446.

10. Heerspink HJL, Stefansson BV, Chertow GM, Correa-Rotter R, Greene T, Hou FF, Lindberg M, McMurray J, Rossing P, Toto R, Langkilde AM, Wheeler DC; DAPA-CKD Investigators. Rationale and protocol of the Dapagliflozin And Prevention of Adverse outcomes in Chronic Kidney Disease (DAPA-CKD) randomized controlled trial. Nephrol Dial Transplant. 2020 Feb 1;35(2):274-282.
11. Wheeler DC, Stefansson BV, Batiushin M, Bilchenko O, Cherney DZI, Chertow GM, Douthat W, Dwyer JP, Escudero E, Pecoits-Filho R, Furuland H, Górriz JL, Greene T, Haller H, Hou FF, Kang SW, Isidto R, Khullar D, Mark PB, McMurray JJV, Kashihara N, Nowicki M, Persson F, Correa-Rotter R, Rossing P, Toto RD, Umanath K, Van Bui P, Wittmann I, Lindberg M, Sjöström CD, Langkilde AM, Heerspink HJL. The dapagliflozin and prevention of adverse outcomes in chronic kidney disease (DAPA-CKD) trial: baseline characteristics. Nephrol Dial Transplant. 2020 Oct 1;35(10):1700-1711.
12. Wheeler DC, Stefánsson BV, Jongs N, Chertow GM, Greene T, Hou FF, McMurray JJV, Correa-Rotter R, Rossing P, Toto RD, Sjöström CD, Langkilde AM, Heerspink HJL; DAPA-CKD Trial Committees and Investigators. Effects of dapagliflozin on major adverse kidney and cardiovascular events in patients with diabetic and non-diabetic chronic kidney disease: a prespecified analysis from the DAPA-CKD trial. Lancet Diabetes Endocrinol. 2021 Jan;9(1):22-31
